# Semi-Supervised Training for Positioning of Welding Seams

**DOI:** 10.3390/s21217309

**Published:** 2021-11-03

**Authors:** Wenbin Zhang, Jochen Lang

**Affiliations:** School of EECS, University of Ottawa, Ottawa, ON K1N 6N5, Canada; wzhan133@uottawa.ca

**Keywords:** welding seam, semi-supervised learning, localization

## Abstract

Robotic welding often uses vision-based measurement to find the correct placement of the welding seam. Traditional machine vision methods work well in many cases but lack robustness when faced with variations in the manufacturing process or in the imaging conditions. While supervised deep neural networks have been successful in increasing accuracy and robustness in many real-world measurement applications, their success relies on labeled data. In this paper, we employ semi-supervised learning to simultaneously increase accuracy and robustness while avoiding expensive and time-consuming labeling efforts by a domain expert. While semi-supervised learning approaches for various image classification tasks exist, we purpose a novel algorithm for semi-supervised key-point detection for seam placement by a welding robot. We demonstrate that our approach can work robustly with as few as fifteen labeled images. In addition, our method utilizes full image resolution to enhance the accuracy of the key-point detection in seam placement.

## 1. Introduction

The use of industrial robots in welding is essential for automation or in hazardous and poor working environments. In general, various types of optical measurements are used to control the trajectory of the robot path for seam tracking [1]. Vision-based measurement [2] can be utilized to recognize and find the position of welding creases to define the weld paths [3]. Xu et al. [4] describe how to mount a camera above a welding torch tip. Vision-based measurement is also used for defect detection of weld beads [5].

**Figure 1:**
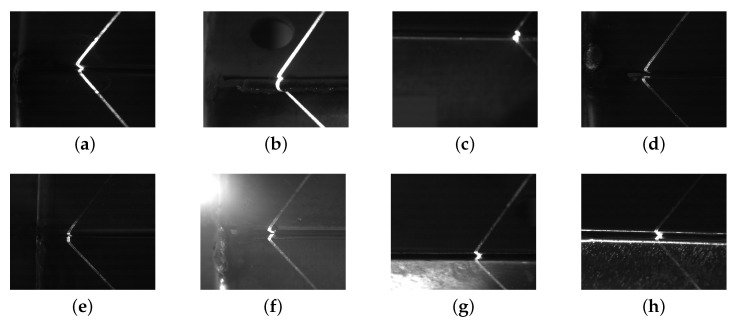
Variation of *valid* images of the weld seam location captured by the welding robot: (**a**) Expected configuration, (**b**) bent edge, (**c**) blurred location, (**d**) large gap, (**e**) small intersection, (**f**) overexposed image, (**g**) unexpected reflection and (**h**) line noise. Our dataset also contains *invalid* images which do not show the location for the weld seam to be placed at all.

Structured light sensing for welding seam tracking is one of the widely used techniques in robotic welding [6]. Images captured by the structured light sensors are less affected by the intensity of the lighting in the welding process than passive vision sensors [7] but nevertheless, different types of metal sheets produce varied light reflection, e.g., stainless steel produces strong specular reflections of the laser light. There is also a lot of noise in the images due to irregular surfaces and due to the image background in the industrial environment as can be observed in the images captured by the camera of the system investigated in this paper (see Figure 1). Existing machine vision systems are successfully applied in industry and produce satisfactory results in many cases. However, these approaches lack robustness [3] in face of variation in imaging conditions, changes in materials to be welded, and geometry changes of the welding setup. This a common scenario with legacy measurement systems in manufacturing as in the case of the robotic welding system investigated in this paper.

The use of supervised deep learning promises to localize the weld position much more robustly and more accurately in varied imaging conditions [7] and has been applied to eye-hand calibration [8,9], weld seam tracking [6] and weld quality control [10,11,12]. However, supervised deep learning only works well with a large number of annotated samples [13]. The annotation challenge is to precisely specify the desired seam location for a whole dataset. It is very labor intensive [14] and may even require welding expertise, and in addition, multiple annotators may differ in their choice of seam positioning. In this paper, we instead propose the use of semi-supervised regression (SSR) to reduce the cost of annotation and the negative impact on accuracy by poorly annotated examples. SSR has not been broadly investigated and most of the common semi-supervised classification (SSC) techniques are hard to apply to regression problems [14,15].

**Figure 2:**
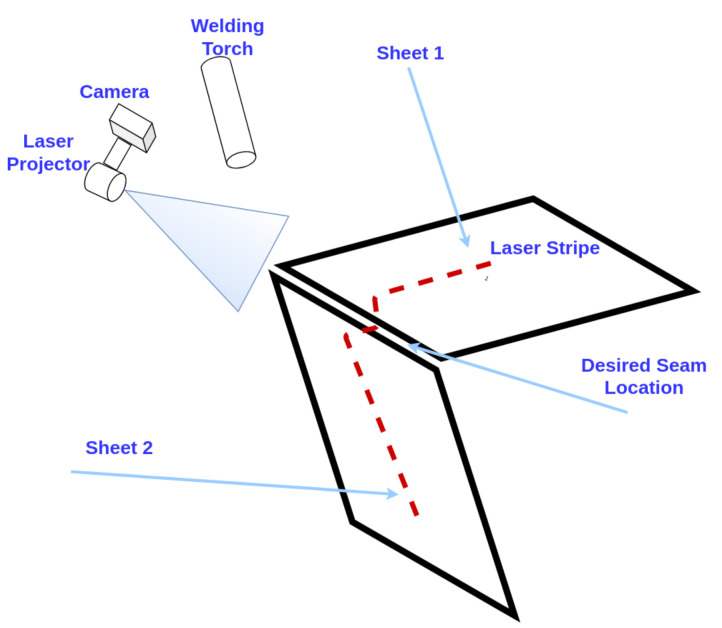
Welding robot configuration. The kinematic relationship between image and welding torch is calibrated.

The specific vision-based measurement problem addressed in this paper is to determine a keypoint in an image that enables a calibrated welding robot to place a seam at the corner of an electrical enclosure (see Figure 2). The electrical enclosure is manufactured from sheet metal. At present, there exists a machine vision system which finds the keypoint by analyzing the laser lines based on assumptions of their relative geometry in the images. The system fails if the image of the laser stripe is blurred, unexpected specular reflection are present in the images or the geometry is not as expected. We use deep learning in order to increase the robustness of the measurement process given geometric variations in the metal bending and when different types of metals are used. In order to apply supervised deep learning, large amounts of images with a label for the exact position of the expected weld joint would be needed in our application. Labeling keypoints in images is very time intensive and error-prone due to a highly repetitive process which needs to be performed with subpixel precision. Therefore, we develop a novel approach for automatic positioning of welding seams on sheet metal enclosures by semi-supervised deep learning with as few as fifteen manually labeled images.

The general idea of our novel semi-supervised approach is to utilize two discriminators (see Section 3.3) focusing on different aspects to validate predictions on unlabeled data. These validated pseudo-labeled data can mix with labeled data in order to retrain our two-stage heatmap generator (see Section 3.2). This process can be run repeatedly until no more improvements can be achieved by adding pseudo-labeled data (see Section 3.4).

Our main contributions are: A semi-supervised training method for a vision-based measurement task that is successful with very few hand-labeled examples; a method to increase the precision of keypoint localization in a two stage network; and a novel generator-discriminator architecture that enforces solution constraints sequentially.

## 2. Related Work

### 2.1. Machine Learning in Welding

Machine learning is increasingly applied to vision-based measurement in welding. Zhang et al. [16] describe a structured light setup on a robot which uses a Hidden Markov model for weld line detection and tracking. Yang et al. [10] use a Convolutional Neural Network (CNN), a simplified Yolo v.3 network [17], for welding defect detection after localizing the weld bead. Normal maps of welding studs are fed to an hour-glass style CNN to create a heat-map for the 2D-localization of keypoints on the stud by Liu et al. [11]. Wang and Shen [12] find the welding zone of water-cooled pipes in radiographic images by semantic segmentation with a specifically designed attention mechanism in their CNN. A CNN-based pixel-to-point module is designed by Zou and Lan [8] for finding weld points in the calibration of a robot laser vision system using reinforcement learning. While both traditional machine learning and deep learning techniques have been applied in vision-based measurement systems for welding, we use U-Net [18] as the baseline network for our heatmap generator which remains a common choice for pixel-to-pixel image tasks such as localization, and VGG-16 [19] as a classifier architecture for our discriminators.

### 2.2. Keypoint Localization

Finding the position of the seam point is a form of keypoint localization. Keypoint localization has been considered in numerous computer vision and vision-based measurement tasks. Newell et al. [20] proposed a Stacked Hourglass Network for landmark detection in human pose estimation. Yang et al. [21] applied such a network design to facial landmark detection. Law and Deng [22] integrate an hourglass network in their CornerNet for the detection of the bounding box for object detection. Their corner pooling enables the localization away from the object features inside the box. State-of-the-art performance on keypoint detection in human pose estimation have been obtained with high-to-low resolution parallel networks in HRNet [23]. In general, coordinate regression can be used to estimate keypoints, but recent methods often use heatmap regression [20,21,22,23,24].

In human landmark estimation, it is often necessary to find many landmarks on RGB images. As a result, these models use numerous network layers in order to derive powerful feature maps from input images. In particular, some models stack multiple sub-networks together to form a much deeper network [20,21] which is computationally costly. However, in order to train these networks, the size of the input has to be down-sampled to a particular size (e.g., 64×64) to feed into the network [20,23,25] due to GPU memory size. This common preprocessing causes the networks to miss useful local features [26]. In contrast, our objective is to localize a single landmark from each image precisely and our dataset contains only gray-scale images. We cannot obtain the desired localization precision if we apply previously mentioned methods. However, instead, our heatmap generator can output much more accurate results by using high resolution input images but requires only a relatively light-weight network.

### 2.3. Semi-Supervised Learning

Semi-supervised learning [27] uses both labeled and unlabeled samples, typically assumed to be sampled from similar distributions. In deep learning, various semi-supervised methods have been pursued including pseudo-labels [28], generative models using auto-encoders [29] and generative adversarial networks [30,31], and teacher-student networks [32]. Recent methods work by exploiting consistency regularization, entropy minimization and generic regularization [33].

Unsupervised data augmentation [34] is a recent consistency regularization approach. It applies data augmentation on unlabeled data and enforces the consistency between the augmented unlabeled sample and the sample itself. Interpolation consistency training [35] instead uses interpolation between two unlabeled samples to enforce the prediction of mixed ‘fake’ labels. In S4L [36], which is a self-supervised semi-supervised learning method, Zhai et al. train their models on an auxiliary task of predicting rotations simultaneously with classifying images. However, the above semi-supervised methods target only classification, and it is hard to see how to extend these methods to image regression problems. In machine learning, regression and classification are distinguished by the output of the prediction task where regression predicts a quantitative output, while classification predicts a categorical or qualitative output [37]. We solve an image regression problem effectively in a semi-supervised manner.

There are some prior self-supervised methods for facial keypoint detection. Dong et al. [38] utilize a differentiable Lucas-Kanade [39] filter to compute a registration loss as supervision to improve the precision of landmark detectors on both images and videos. The method computes optical flow registration in the forward pass, and back-propagates gradients that encourage temporal coherency in the detector. Our data consists however only of single image input. Our approach is closest to Dong and Yang [32] who propose a self-paced learning algorithm for facial landmark detection from partially labeled samples. The method uses two student networks to generate pseudo-labeled keypoints which are than filtered by a teacher to only accept qualified pseudo-label for further training of the students. However, the labeled samples to train the two students network initially have to be independent, which means more labeled samples are required for supervised training as for just a single student network. In contrast, our network share the same number of labeled samples to train both a heatmap generator and two discriminators as teachers. By this architecture, our network requires no additional labeled data. More details on student-teacher networks for different knowledge distillation tasks can be found in a recent survey [40].

Honari et al. [41] propose a sequential method consisting of three phases to improve landmark localization with semi-supervised learning. They train a CNN based detector with ground-truth landmarks, which is further trained as an intermediate step for a different but related task and in the final phase, an equivarient transformation constraint is used on the input images and the heatmap of the keypoints. Their solution requires images of the application domain annotated for a related tasks which does not exist for initial welding point localization. Earlier, Ukita and Uematsu [42] used labeled and weakly-labeled human poses in different sports to predict human landmarks. Yao et al. [43] design a semi-supervised keypoint detection method for multi-view reconstruction utilizing an epipolar constraint from stereo vision. Kumar and Chellappa design S2LD [44] for semi-supervised learning of facial landmarks in small images based on a multiple generator and discriminator networks design. Their solution is specific for human faces and assumes that annotated examples for high-resolution images exist. Cho et al. [45] address detection in medical images during domain adaption but related to our work, they use a heatmap generator with a Gaussian peak in a generator-discriminator like network. They do not address lack of precision labels as in our work.

The work by Moskvyak et al. [14] considers the task of semi-supervised learning for keypoint localization for wild animals and as our work is motivated to reduce the labeling effort in annotating specific dataset. Their method uses three constraints during semi-supervised training with few labeled and many unlabeled examples. They adapt the transformation consistency loss of Honari et al. [41] into a transformation equivarient constraint for the actual heatmap and a transformation invariant constraint for the labels. Their main contribution is a classification loss based on the features of a keypoint, i.e., across different heatmaps a mapping between the semantic keypoint and its class (e.g., the beak of birds) is enforced. The loss therefore is based on the category for different keypoints and does not apply to single keypoint detection as in our task.

## 3. Proposed Method

### 3.1. Overview

**Figure 3:**
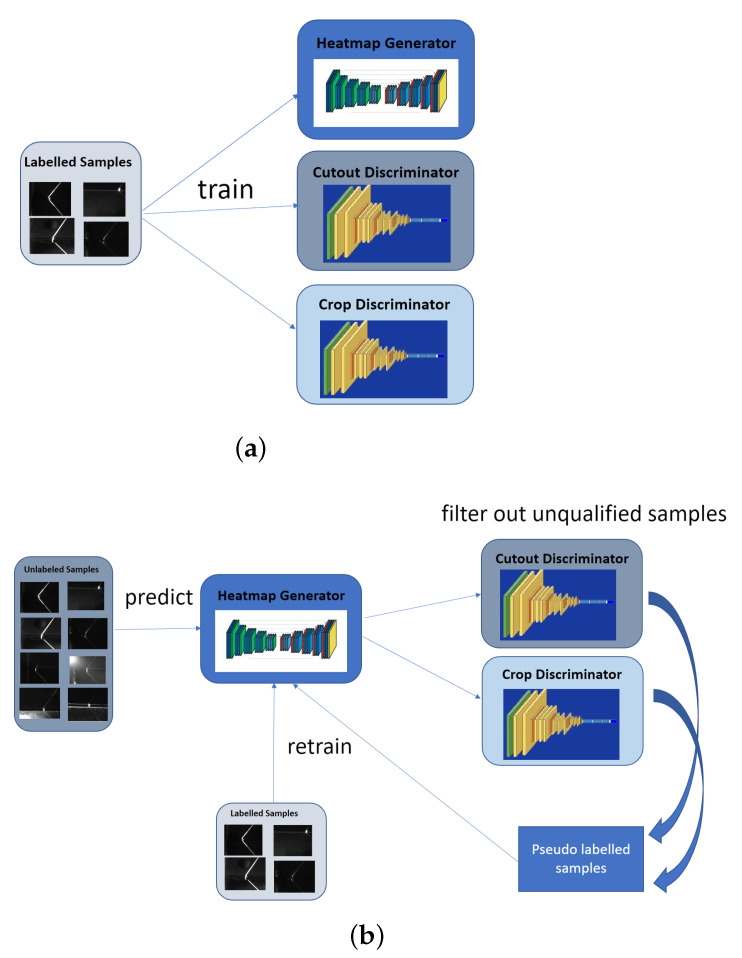
Two phase semi-supervised training strategy of heatmap generator and double discriminator for our keypoint detector. (**a**) Phase 1: Independent supervised training for the heatmap generator and two discriminators. (**b**) Phase 2: Semi-supervised training of the heatmap generator with the help of two discriminators from Phase 1.

We propose a generator and discriminator structure similar to a GAN [46] but employ a different training strategy to find qualified pseudo-labels in semi-supervised learning. The training of our method does not require solving a challenging minmax optimization. Our discriminator networks aim to filter out the best predictions in unlabeled images from the output of the generator. These qualified predictions are used as pseudo-labeled data during the semi-supervised training phase to retrain the generator for the next training step (see Figure 3).

### 3.2. Heatmap Generator

We regress the heatmap of the keypoint. A heatmap representation can achieve higher accuracy with small datasets and shallow models [47] than direct regression of the coordinates. For supervised training, the ground truth coordinates need also be converted to a heatmap where the location is *hot* if close to the ground truth coordinates. There are many ways to create these heatmaps. In our approach, we pick an isotropic Gaussian kernel

(1) $$\;Gaussian\left(\sigma \right)=\frac{1}{2\pi \sigma^{2}}e^{\frac{\sum_{m=0}^{1}{\left(p_{k,m}-c_{m}\right)}^{2}}{2\sigma^{2}}},$$

as the method to generate heatmap labels from coordinates for each training image, where $c_{0}$ and $c_{1}$ are the horizontal and vertical coordinates of the keypoint, respectively, and $p_{k,0}$ and $p_{k,1}$ are the horizontal and vertical pixel coordinates of the heatmap, respectively. The radius of the Gaussian kernel is determined by σ which we set to 0.6 pixels in all experiments.

**Figure 4:**
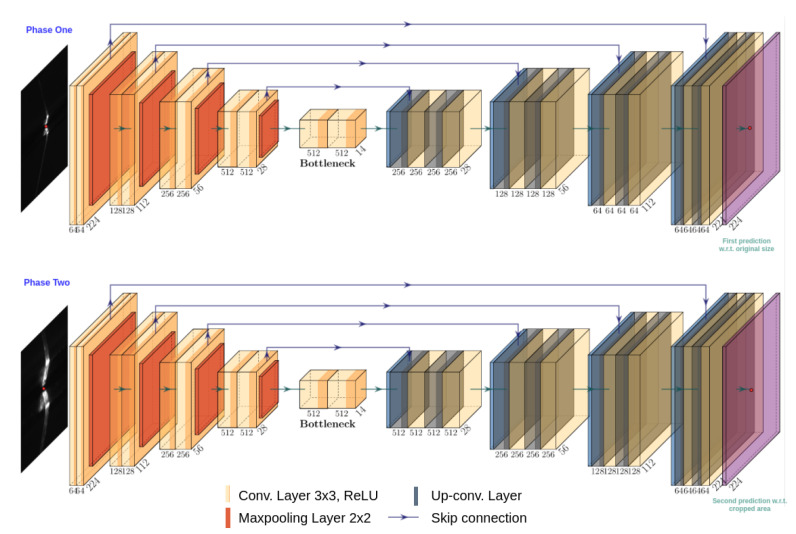
Stages 1 and 2 of the heatmap generator network. (The red dot is only for illustration and is missing in the actual images.)

We base the architecture of the generator on U-Net by Ronneberger et al. [18]. We use rectified linear units as the nonlinear activations in the convolutions and batch normalization layers. Each downsample block contains two 3×3 convolution layers, two batch normalization layers, two activation layers, and one 2×2 pooling layer. Each upsampling block contains a bilinear upsampling layer, two 3×3 convolutional layers, 2 batch normalization layers, and 2 two activation layers. The input of the network is a 224×224 single channel grayscale image and the output of the network is single channel heatmap of the same size. Figure 4 show the details of the generator.

#### Zoom-In Attention Area

Downsampling images to reduce memory requirements causes important information loss which affects the precision of the final prediction. If we increase the resolution of the input image directly, the number of parameters and the memory requirements increase and may require more powerful hardware. To overcome this problem, we take inspiration from the facial keypoint detector by Chandran et al. [48] that detects region of interests in a proxy image to guide high resolution crops from the original high resolution input image. Our strategy is to use a two stage heatmap generator that can zoom-in to the relevant area of the original full resolution image without increasing the parameters significantly but also achieves better precision. This detection approach consists of two identical networks, each one with its own parameters. The first network in Stage 1 estimates a coarse resolution heatmap by taking a downsampled image as input. The second network in Stage 2, estimates a heatmap at the full resolution of the original higher resolution image but only in a cropped area centered at the peak in coarse resolution heatmap output from Stage 1. The second stage network acts as a refinement network for the first stage prediction.

**Figure 5:**
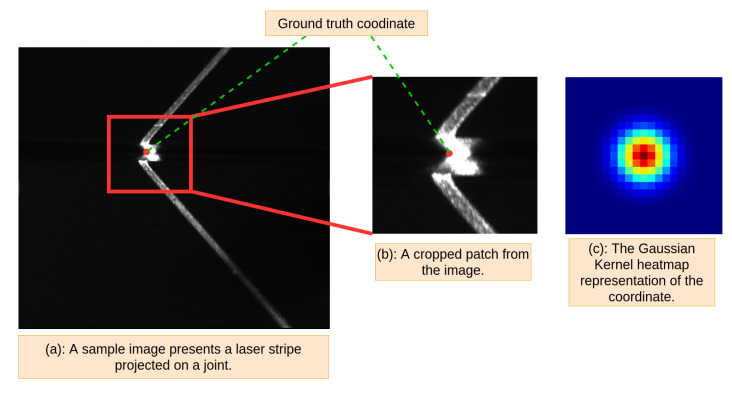
(**a**) A sample image of a joint with laser stripe projection. (**b**) A zoom-in area from the sample image. (**c**) The Gaussian heatmap representation of the ground truth coordinate.

We write the heatmap generator network with stages s∈{1,2} with $\psi_{s}$ be the generator function and trainable parameters $\theta_{s}$ for each stage. Thus, the output of the heatmap generator can be written as

(2) $$h_{s}=\psi_{s}\left(x_{s};\theta_{s}\right)$$

where $x_{1}$ is the downsampled input image and $x_{2}$ is a full resolution zoom-in of the input image according to the prediction $h_{1}$. Outputs of networks $h_{1}$ and $h_{2}$ are in the form of heatmaps where the network predicts the probability of the desired keypoint at each and every pixel. Then the $L_{2}$ loss is

(3) $$Loss=arg\;min_{\theta }\sum_{s=1}^{2}\left(\frac{1}{K}\sum_{k=0}^{K-1}\|h_{s,k}-\psi_{s}\left(x_{s,k}|\theta_{s}\right){\|}_{2}^{2}\right)$$

where *K* is the number of pixels in the last layer of the network. We use the same architecture for both coarse and fine stage networks (see Figure 4). We train both stages of the heatmap generator by minimizing the $L_{2}$ distance between the prediction and ground truth heatmap. In order to train the second stage of the generator, we crop an area from the original image centered on an uniform random offset from the groundtruth coordinate. Figure 5 show an example of the input image with groundtruth, zoom-in attention area, and heatmap representation.

### 3.3. Double Discriminators

Cutout or masking removes part of an image and is often applied as a regularization technique [49] but we use a cutout and a crop discriminator. Our cutout discriminator evaluates keypoint predictions by deciding if an image with a masked out area does no longer contain enough features to locate the keypoint. Similarly, the crop discriminator also evaluates keypoint predictions but in a complementary manner to the cutout discriminator by deciding if a crop still contains the keypoint.

**Figure 6:**
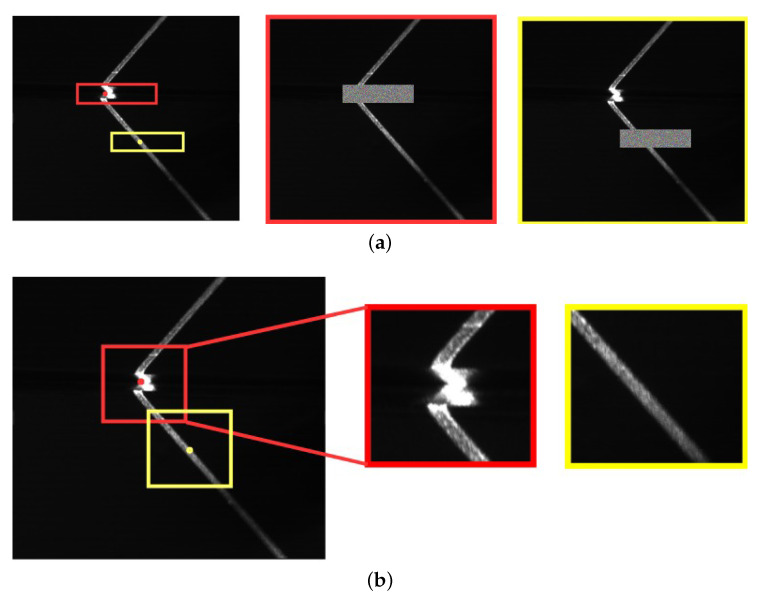
Modification of input images for the discriminator networks based on the keypoint $c_{m}$ and a random offset $\Delta d_{m}$. Red frames correspond to positive samples while yellow show negative samples. (**a**) Replacing cutout area with randomness. Red frames correspond to positive samples while yellow show negative samples. (**b**) Cropping attention area centered by the prediction. Red frames correspond to positive samples while yellow show negative samples.

**Figure 7:**
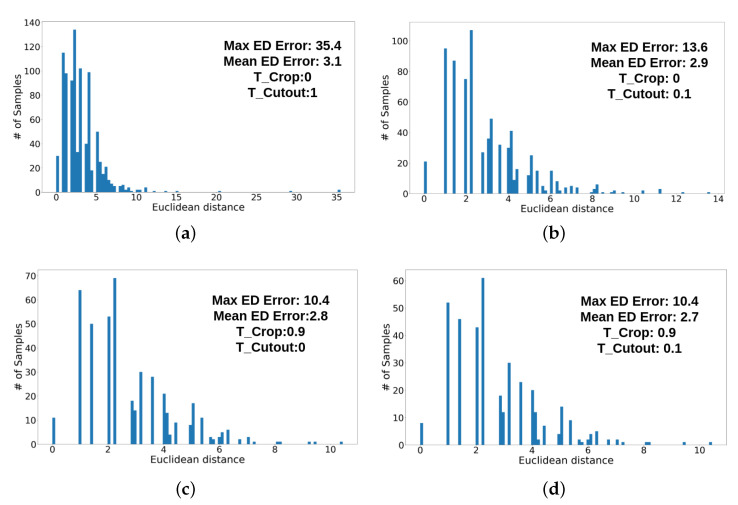
Error histograms of predictions from the heatmap generator while tightening thresholds of the cutout and crop discriminators. The x-axis indicates the Euclidean distance between the prediction and the ground truth. The y-axis indicates the number of predictions. Please note the different scales of the x-axis. (**a**) All predictions ($t_{cutout}=0.0,t_{crop}=0.0$). (**b**) $t_{cutout}=0.1,t_{crop}=0.0$. (**c**) $t_{cutout}=0.0,t_{crop}=0.9$. (**d**) $t_{cutout}=0.1,t_{crop}=0.9$.

We adapt the feature extraction layers of VGG-16 [19] and append two fully connected layers for each of our two discriminators. Each discriminator network outputs a single confidence score for the input image, which is either a cropped area from the original image, or an image with a cutout area replaced by some random grayscale value. Figure 6 shows examples of modified images as input to the cutout and crop discriminator, respectively. Both discriminators examine the quality of the prediction from the heatmap generator. Figure 7a depicts the Euclidean distance distributions of 927 predictions from the generator. Most of the predictions have an error range from 0 to 5 pixels, but some outliers have an error larger than 10 and in particular, the maximum Euclidean error distance is around 35 pixels. Those outliers may drastically reduce the accuracy of the generator if they would be used as pseudo labels for the next training step. Figure 7b shows that we can apply the cutout discriminator to filter out some outliers. As the result, both the maximum Euclidean error and mean Euclidean error can be reduced. On the other hand, the error also can be reduced if we only use the crop discriminator to filter out outliers (Figure 7c). Figure 7d shows utilizing both, cutout and crop discriminators to detect and filter out outliers. Consequently, we can reduce the maximum Euclidean error and the mean Euclidean error to 2.7. Figure 7 shows that when we tighten the thresholds on the respective confidence scores $t_{cutout}$ and $t_{crop}$, an increasing number of outliers can be eliminated. The details of each discriminator are explained below.

#### 3.3.1. Cutout Discriminator

Inspired by [49], we cutout a fixed size area from the image centered according to the predicted keypoint from the heatmap generator (see Figure 6a). The idea of this discriminator is to transfer the regression problem to a binary classification sub-problem. The network is to test if the cutout has removed the area of the keypoint based on the features left in the image after cutout. If the heatmap generator predicts the keypoint location correctly, than cutout of the predicted area will result in an image that does not contain features corresponding to the key area anymore. Images without the keypoint are classified as negative by the cutout discriminator. On the other hand, if the image after cutout still contains enough features for localizing the keypoint, then it will be marked positive.

We gain the extra benefit of being able to create additional label data to train the discriminator by using various differently placed cutouts with a fixed amount of labeled data. We add some uniform random variable $\Delta d_{1},\Delta d_{2}$ to the ground truth coordinate. Then evaluate the Euclidean Distance (ED) between the original and modified coordinate and assign a label according to a maximum distance from the keypoint $d_{max}$,

(4) $$L_{cutout}=\left\{\begin{array}{cc} 1 & \mathrm{if}\;\sqrt{\sum_{m=0}^{1}{\left(\Delta d_{m}\right)}^{2}}\geq d_{max}\\ 0 & \mathrm{if}\;\sqrt{\sum_{m=0}^{1}{\left(\Delta d_{m}\right)}^{2}}<d_{max} \end{array}\right..$$

The maximum distance must be set large enough to remove all features associated with the keypoint. The replacement value for the cutout area is a hyper-parameter, and we found that using all zeros or some random value work equally well.

#### 3.3.2. Crop Discriminator

The crop discriminator has a similar structure as the cutout discriminator. However, the input of the network uses only a small cropped area that contains the most important features for the keypoint (see Figure 6b). Based on the cropped area, the crop discriminator categorizes the cropped area as containing enough features of the keypoint or not and hence the label is

(5) $$L_{crop}=\left\{\begin{array}{cc} 0 & \mathrm{if}\;\sqrt{\sum_{m=0}^{1}{\left(\Delta d_{m}\right)}^{2}}\geq d_{max}\\ 1 & \mathrm{if}\;\sqrt{\sum_{m=0}^{1}{\left(\Delta d_{m}\right)}^{2}}<d_{max} \end{array}\right..$$

During training, the cropped area is centered on the ground truth coordinates plus a random offset $d_{m}$ in the horizontal and vertical direction. selected coordinates can be labeled as invalid class. Random offsets of the cropped area by a distance larger than the threshold $d_{max}$ from the groundtruth coordinate are negative samples.

As each discriminator has its own strategy to extract different types of features, it has the potential to capture different outliers. As a result, cutout and crop discriminator can supplement each other. By using two discriminators, we create a classification task as an intermediate step when we try to solve the overall regression problem. Formally, the loss function of both discriminator networks are

(6) $$Loss\left(D\left(x|\theta \right),L\right)=\frac{1}{N_{D}}\cdot \sum_{i}^{N_{D}}\left(y^{i}log\left(D\left(f\left(x^{i}\right)\right)\right)+\left(1-y^{i}\right)log\left(1-D\left(f\left(x^{i}\right)\right)\right)\right).$$

where *f* indicates a modification function on the sample image *x* using the random offset $\Delta d_{m}$ that outputs positive and negative images labeled according Equations (4) and (5), respectively. Where $logD(f\left(x^{i}\right))$ refers to the probability that the positive sample is correctly classified. In contrast, $log(1-D\left(f\left(x^{i}\right)\right))$ refers to the probability that the negative sample is correctly classified and $y^{i}$ is the groundtruth for sample *i* where $y^{i}=1$ for positive and $y^{i}=0$ for negative samples. The number of training images for the two discriminators is $N_{D}$.

### 3.4. Overall Semi-Supervised Algorithm

We propose a two-phase framework for learning from unlabeled samples. In the first phase, we train our networks by a limited number of labelled samples which is shown in Figure 3a. In the second phase, we retrain the heatmap generator with pseudo labelled samples which are obtained by filtering out unqualified predictions as shown in Figure 3b. Our semi-supervised algorithm aims to progressively improve the performance of the heatmap generator. A key advantage of our algorithm is that three networks (generator and two discriminators) can share the same labeled data to train in Phase 1 since they are independent networks, and each network has a unique structure and goal. Moreover, during the process of training the two discriminators, we can generate additional training samples by random cutout and cropping from the original labeled images. This training strategy on discriminators reduces the required minimum number of labeled samples. The algorithm is detailed in Algorithm 1 where $x^{l}$ indicates a labeled image and $x^{u}$ indicates an unlabeled image. The training algorithms uses $n_{l}$ labeled images and $n_{u}$ unlabeled images. The influence of the hyper-parameters $t_{cutout}$, $t_{crop}$ are illustrated in Figure 7.
**Algorithm 1.** Overall Algorithm.**Require:** Labeled data $L=\{(x_{i}^{l})|1\leq i\leq n_{l}\}$
**Require:** Unlabeled data $U=\{(x_{i}^{u})|n_{l}+1\leq i\leq n_{u}+n_{l}\}$**Require:** Heatmap generator *ψ*_1,2_ with *θ*_1,2_**Require:** Discriminator *D_cutout_* with *θ_cutout_***Require:** Discriminator *D_crop_* with *θ_crop_*    Initialize *θ*_1,2_ by minimizing Equation (3) on L    Initialize *θ_cutout_* and *θ_crop_* by minimizing Equation (6) on L    Threshold $t_{cutout}\leftarrow 0\ldots 1$    Threshold $t_{crop}\leftarrow 0\ldots 1$    J← maximum # steps    **for**
*j* = 1 to *J*
**do**        Predict $y_{1,2}^{j}$ on U using *ψ*_1,2_, and denote U with its pseudo labels as U’        Compute the confidence of each prediction $y_{1,2}^{j}$ for U’ using *D_cutout_* and *D_crop_*, respectively        $L^{\prime }\leftarrow$ qualified samples from U’ determined by *D_cutout_* and *D_crop_* with *t_cutout_* and *t_crop_*, respectively.        Retrainby *θ*_1,2_ on $L^{j+1}=L\cup L^{\prime }$ by minimizing Equation (3)    **end for**    **return** Generator *ψ*_1,2_ with optimized parameters *θ*_1,2_

## 4. Experiments and Results

After a description of the data that has been acquired at the industrial site by our collaborator, we compare our heatmap generator with state-of-the-art supervised keypoint detection methods and also evaluate the effectiveness of the second stage of our heatmap generator. Then we investigate how many labeled images are required for our semi-supervised training strategy and how robust the results are under random selection of labeled images. We include a comparison with TS3 by Dong and Yang [32] which is the closest semi-supervised approach to ours. Finally, we provide a run-time analysis of our methods during forward prediction.

### 4.1. Data Description

We have obtained our data from EDI Inc. (St. Petersburg, FL, USA) which manufactures electrical enclosures. All the images are grayscale and of size of 1280×1024. Figure 1 shows example images from the data set. The laser stripe projection is clearly visible. Different types of joint shapes generate different stripe patterns due to noisy reflections, varied exposure and different metals. All images are taken from the same angle and orientation which means the projection of the laser stripe is always towards a fixed direction for every image (see Figure 2). We have 88,231 unlabeled images and we have hand-labeled 8342 images for our experiments. Depending on the specific experiment, we utilize fewer of the labeled images to investigate how many labeled images are required by our method. All labeled images are annotated by the horizontal and vertical pixel coordinates of the starting point for the welding seam which is the keypoint to be identified by our method. Unlabeled images include invalid images which we define as images where the location for the seam is not visible. These images occur at a ratio of approximately 1:9 and are easily identified by the discriminators during semi-supervised training.

In order to demonstrate the effectiveness of our semi-supervised method, we train our network with only a small number of labeled samples. We conduct our experiments with 200, 100, 50, 20 and 15 randomly selected labeled samples.

### 4.2. Experimental Settings

#### 4.2.1. Training the Heatmap Generator

In training the heatmap generator, we apply the Adam optimizer with a learning rate of 0.0001. For stage one (S = 1) of the heatmap generator, we resize the 1280 × 1024 image to 224 × 224, first. Zoom-in during the second stage (S = 2) also uses a 224 × 224 input. We find that using a batch size of 10, 15, and 20 leads to the same performance. We use 200 epochs for training the generator with the maximum batch size for the GPU memory.

#### 4.2.2. Training the Two Discriminators

The discriminator networks take images of 224 × 224 as input and they output a scalar value. We use the Adam optimizer with a learning rate of 0.0001 to train the two discriminators. We train the discriminators with the labeled training and validation images at the beginning of the supervised training step. We use data augmentation as described in Section 3.3 where we generate seven images for the positive class and eight images for the negative class for the cutout discriminator, and one image each for crop discriminator. We use a threshold $d_{max}=300$ pixels. The training is stopped when the accuracy reaches over 90%. The cutout discriminator typically needs 100–150 epochs, the crop discriminator needs 50–100 epochs.

#### 4.2.3. Metric and Evaluation

We pick Mean Square Error (MSE) as our objective function for the heatmap generator as it can produce the closest heatmap to the target heatmap. Where MSE for our two stage generator is defined as

(7) $$L_{MSE}\left(\psi_{2}\left(x|\theta_{2}\right)\right)=\frac{1}{K}\sum_{k=1}^{K}\|h^{gt}-h_{2}{\|}^{2}$$

where the heatmap $h_{2}$ is defined in Equation (2). The peak value of the heatmap $h_{2}$ corresponds to the predicted keypoint but the other pixel values of the heatmap are of no importance in our application. Appropriately, to quantitatively evaluate the performance of the generated heatmap, we use the Euclidean Distance (ED) of the peak from the groundtruth coordinate to estimate the actual quality of the prediction. Hence, we report the Mean Euclidean Distance (MED) error.

### 4.3. Results and Discussion

#### 4.3.1. Comparison with Supervised Learning

Our first set of experimental results compare our two-stage heatmap generator with other keypoint detection methods. The comparators in Table 1 are: Stacked Hourglass Network [20], a Simple Baseline [50], and HRNet [24]. We include the Stacked-Hourglass as a classic method. SimpleBaseline is a reasonable choice for datasets that lack the challenges of multi-pose detection and of significant scaling as in our task. HRNet remains the foundation for many of the best performing bottom-up approaches for human keypoint detection. We train our proposed network and the supervised comparison methods with 7000 labeled images for training and 415 for validation. We evaluate the performance of all methods on 927 testing images. We observe that our lightweight network with its two-stage heatmap generator is the best fit for the task at hand, yielding the lowest error of all methods considered. The MED error is about 4 pixels less with our network than with the Stacked Hourglass Network with any of one, two and eight stacked hourglass models. We found HRNet to perform similar to the Stacked Hourglass method, while the Simple Baseline performed worse. Our method is not designed and is not expected to be competitive for human landmark or keypoint detection, as such methods must model the relationship between keypoints which is not part of our task.

We have also included a comparison using our heatmap generator without its second stage for zoom-in. We can see that the second stage improves the result by about 0.4 pixels. Using all labeled samples in the training set is sufficient for training our network. This can be seen as there is no significant improvement by applying our semi-supervised technique for three iterations. Next we will report results with a reduced number of samples that demonstrate the ability of our semi-supervised technique to achieve better results than state-of-the-art supervised methods with significantly fewer labeled images.

#### 4.3.2. Comparison with Different Number of Labeled Data

**Figure 8:**
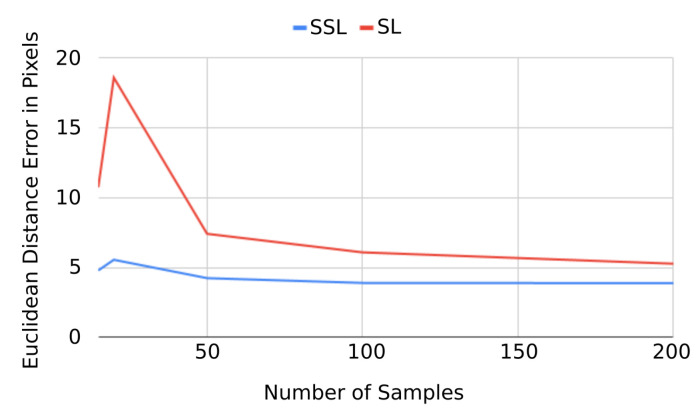
Performance of semi-supervised learning (SSL) vs. supervised learning (SL).

We conduct experiments with different number of samples selected randomly from all labeled data and report test results on the 927 testing samples. The test samples are strictly used for testing and our methods uses only the training data for fitting. The details of the experimental results are shown in Table 2. In the first group of data, we randomly select 200 labeled samples and separate them by ratio of 9:1 for training and validation, respectively. The MED error is 5.278 pixel after supervised learning. By applying our semi-supervised process three times consecutively, the MED error can be reduced to 4.328, 3.968 and 3.885, respectively. We then reduce the number of labelled data to 100, 50 and 20, respectively, while keeping the same ratio for training and validation. As expected the error in the supervised step increases with the reduction in the number of labelled samples, however, the semi-supervised steps are able to reduce the error even with just 20 labeled images for training and validation. After three semi-supervised training steps, the error is reduced from a MED of 18.598 to 5.56 which is not quite as low as when using 200 labelled samples but it is still well below the Stacked Hourglass and HRNet methods (cf. with Table 1). Figure 8 summarizes the performance of our SSL approach demonstrating a consistent benefit in terms of error over supervised training independent of number of labelled samples. As to be expected, the benefit of semi-supervised training is largest when the number of labelled samples is small.

#### 4.3.3. Comparison with Random Labeled Data

**Figure 9:**
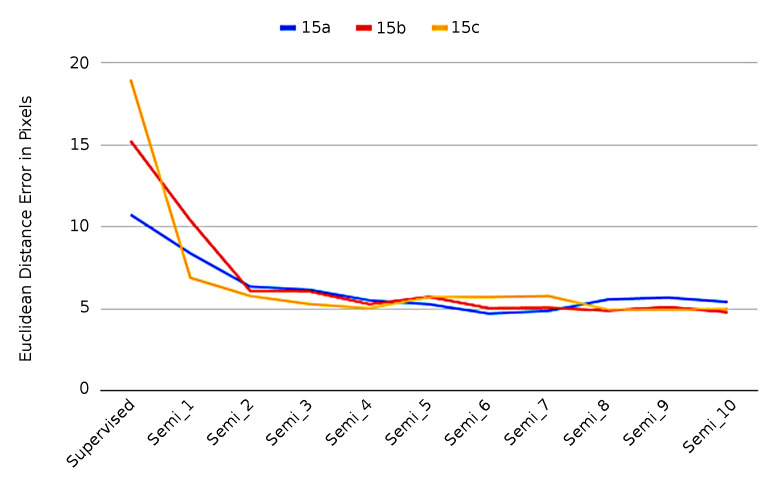
Performance of 3 groups of 15 labeled samples.

We investigate the robustness of our approach with a small number of labeled images by training our semi-supervised method with different randomly selected label images. We randomly pick 3 groups of 15 labeled samples, and split each group randomly into 10 samples for training and 5 samples for validation. We evaluate the performance of each model again with the same testing dataset of 927 samples. The experimental results are shown in Table 3. The same number of labelled samples lead to similar performance after a sufficient number of semi-supervised training steps. We also run 3-fold cross validation on each 15 samples, and we end up with consistent results. Observing the error on the validation and on the testing dataset, we can conclude that there is a positive correlation between the validation loss and the testing error. Figure 9 demonstrates that training a model with the same number of random labeled samples can be expected to lead to similar performance after enough semi-supervised training steps.

The Figure 9 and Table 3 shows that the performance of the heatmap generator can be significantly improved by utilizing the semi-supervised technique. After several semi-supervised iteration, the error drops from supervised learning stage dramatically and constantly. The accuracy of a heatmap generator trained by 15 random labeled samples followed by semi-supervised steps is very close to a generator trained by 7415 labeled samples.

#### 4.3.4. Run-Time

**Figure 10:**
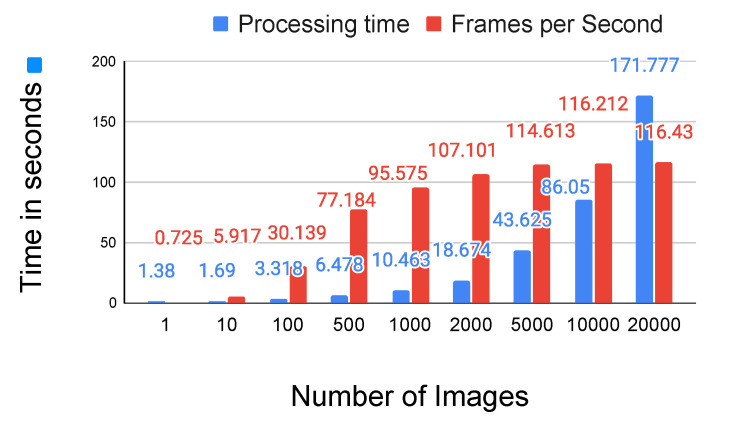
The average runtime of the heatmap generator during prediction. The blue bars are the processing time in seconds and the red bars are the frames per second (FPS) for a particular number of sample images in a batch. The shown result is the average over 10 runs for each batch.

After finishing the training process, the heatmap generator is the only part required for on-line deployment. The average runtime of the heatmap generator over 10 predictions is shown in Figure 10. We made no attempt to reduce the overhead due to initialization which can be seen from the low frame-rate with a small number of samples. As can be seen with 500 or more sample images our method executes faster than video frame rate. The runtime of the heatmap generator during prediction is independent of the number of training images. The number of parameters for each stage of the heatmap generator is 17.27 MB and its FLOPs is 30.66 GMac. All the computations in the test are conducted on a single workstation (Intel Core i7-8700K CPU, 16 GB system memory, NVIDIA Geforce RTX 3080, Pytorch version 1.8.0 and CUDA version 11.1).

#### 4.3.5. Comparison with the State-of-the-Art in Semi-Supervised Methods

**Figure 11:**
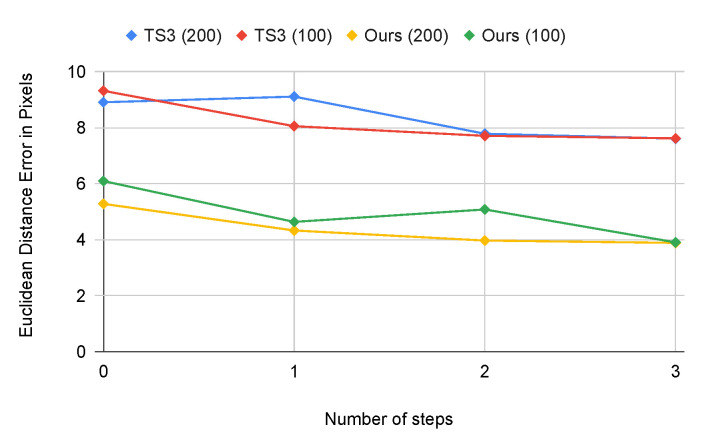
We compare our method with TS3 [32] on our dataset with 200 and 100 labeled images, respectively. We run each model by three semi-supervised iterations. The x-axis indicates the results of four models. The y-axis indicates the Euclidean Distance error.

We compare our method with TS3 by Dong and Yang [32] as shown in Figure 11. As discussed in Section 2.3, most other semi-supervised learning methods are task-specific and cannot be directly applied to our task of single keypoint detection of the initial weld position. TS3 [32] is a state-of-the-art semi-supervised method for partially labeled images for facial landmark detection. We use the implementation and hyper-parameters of the authors. Compared to TS3, our proposed method achieves a lower MED error with 200 and 100 labeled images. TS3 is designed to detect multiple keypoints of a human face while our method localizes only one keypoint. Face images show also likely more variety than is present in our dataset. In order to work with faces TS3 contains two heatmap generators which are both very deep neural networks. We suspect that TS3 with its dual student design is too complex to fit our task and hence is not able to fully exploit the unlabeled images. Compared to TS3, the discriminators in our proposed method are simple to train with only labeled images because of effective data augmentation. The generator is then retrained in the steps of the semi-supervised training. In other words, the proposed method is overall simple to train. Furthermore, during the deployment of the proposed method, the discriminators are not needed and hence the proposed method only needs a single heatmap generator and not two students as in TS3. This improves run-time and reduces memory cost.

## 5. Conclusions

In this paper, a new vision-based measurement method to detect a keypoint for determining the correct placement of a welding seam was introduced. The method uses a neural network architecture that consists of a two-stage heatmap generator and two discriminators. It has been shown that the two-stage heatmap generator can localize the keypoint with a mean Euclidean distance error of just over 3 pixels at video-rates. Our proposed semi-supervised training method is able to work with as few as 15 labeled images due to the two discriminators. The discriminators need only be trained at the beginning on the same labeled images with data augmentation. Then, the discriminators enable our pseudo-labeling approach to utilize a large number of unlabeled images to reach a very competitive mean Euclidean distance error. This makes our proposed method easy to train in new configurations of the welding robot as the manual labeling effort is minimal with just 15 images. We have shown that our novel semi-supervised training approach is both simple and effective, and outperforms a popular semi-supervised comparison strategy on the keypoint detection task at hand. In future work, we like to extend our semi-supervised training approach to multi-keypoint regression problems in industry.

## Figures and Tables

**Table 1:** Comparison with state-of-the-art supervised methods. The number of semi-supervised steps in our method is *J* and the stages of our heatmap generator is S (see Algorithm 1). Given a large number of labeled training images, semi-supervised steps do not improve model fit further. However, zoom-in, i.e., using our two-stage heatmap generator successfully reduces the mean Euclidean distance (MED) error in pixels.

Network	Ours					Others		
	Supervised	*J* = 1	*J* = 2	*J* = 3	Supervised	Stacked Hourglass [20]	Simple Baseline [50]	HRNet [24]
	2 Stage Zoom-in S = 2				S = 1			
Labeled Images	7415	7415	7415	7415	7415	7415	7415	7415
Unlabeled Images	0	88,231	88,231	88,231	0	0	0	0
Input Size	224 × 224	224 × 224	224 × 224	224 × 224	224 × 224	256 × 256	256 × 256	256 × 256
Block Size	224 × 224	224 × 224	224 × 224	224 × 224	n/a	64 × 64	64 × 64	64 × 64
MED	**3.139**	**3.127**	**3.263**	**3.199**	3.545	7.471	8.244	7.401

**Table 2:** Effect of number of labeled samples on testing error (MED in pixels).

#Labeled	#Unlabeled	#Testing	Supervised	Semi-Supervised Steps		
(#Training/#Validation)				*J* = 1	*J* = 2	*J* = 3
200 (180/20)	88,231	927	5.278	4.328	3.968	3.885
100 (90/10)	88,231	927	6.091	4.634	5.078	3.903
50 (45/5)	88,231	927	7.718	6.213	5.256	4.245
20 (18/2)	88,231	927	18.598	8.646	5.560	5.606

**Table 3:** Robustness of testing error (MED in pixels) with 15 labeled samples (10 for training and 5 for validation).

Set	#Labeled	#Unlabeled	#Testing	Supervised	Semi-Supervised Steps							
	(#Training/#Validation)				*J* = 1	*J* = 2	*J* = 3	*J* = 4	*J* = 5	*J* = 6	*J* = 7	*J* = 8
(a)	15 (10/5)	88,231	927	15.252	10.401	6.101	6.063	5.283	5.732	5.040	5.067	4.890
(b)	15 (10/5)	88,231	927	10.756	8.387	6.360	6.164	5.518	5.282	4.713	4.874	5.574
(c)	15 (10/5)	88,231	927	12.853	7.401	6.464	6.392	6.098	5.952	5.748	5.988	5.754

## Data Availability

Not applicable.
